# A multilevel analysis of health-related physical fitness. The Portuguese sibling study on growth, fitness, lifestyle and health

**DOI:** 10.1371/journal.pone.0172013

**Published:** 2017-02-10

**Authors:** Sara Pereira, Peter Todd Katzmarzyk, Thayse Natacha Gomes, Michele Souza, Raquel Nichele Chaves, Fernanda Karina dos Santos, Daniel Santos, Donald Hedeker, José Maia

**Affiliations:** 1 CIFI2D, Faculty of Sport, University of Porto, Porto, Portugal; 2 Pennington Biomedical Research Center, Louisiana State University, Baton Rouge, LA, United States of America; 3 Department of Physical Education, Federal University of Santa Catarina, Florianópolis, Santa Catarina, Brazil; 4 Federal University of Technology-Paraná (UTFPR), Campus Curitiba, Curitiba, Brazil; 5 Department of Physical Education, Federal University of Viçosa, Viçosa, Minas Gerais, Brazil; 6 Department of Public Health Sciences, University of Chicago, Chicago, IL, United States of America; Vanderbilt University, UNITED STATES

## Abstract

This study investigates biological, behavioural and sociodemographic correlates of intra-pair similarities, and estimates sibling resemblance in health-related physical fitness (PF). The sample comprises 1101 biological siblings (525 females) aged 9–20 years. PF components and markers were: morphological [waist circumference (WC) and %body fat (%BF)], muscular [handgrip strength (GS) and standing long jump (SLJ)], motor [50-yard dash (50YD) and shuttle run (SR)], and cardiorespiratory (1-mile run). Biological maturation was assessed; physical activity (PA), TV viewing and socioeconomic status (SES) information was obtained. On average, older and more mature subjects are better performers in all PF components; PA was negatively associated with SR, while SES was negatively associated with SLJ and SR. A pattern was observed in the intraclass correlations (ρ) wherein same sex siblings demonstrate greater resemblance for most PF components (sister-sister: 0.35≤ ρ≤0.55; brother-brother: (0.25≤ρ≤0.60) than brother-sister pairs (BS) (0≤ρ≤0.15), except for %BF (ρBB>ρSS>ρBS), and the 1-mile run (ρSS>ρBS>ρBB). In conclusion, behavioural and sociodemographic correlates play different roles in siblings PF expression. Further, a significant familial PF resemblance was observed with different trends in different sibling types, probably due to variations in shared genetic factors and sociodemographic conditions.

## Introduction

Physical fitness (PF) is a complex construct, broadly described as an individual attribute expressing the efficiency of a varied set of bodily systems and functions to perform work in a wide-ranging set of contexts [1]. It is widely accepted that the multivariate structure of PF varies in terms of its configuration, namely in the expression of its basic components and indicators [2]. Bouchard & Shephard [1] presented a comprehensive definition of health-related PF, composed of several components (morphological, muscular, motor, cardiorespiratory, and metabolic) which has been used in several studies [3–5]. Further, it has been shown to be a consistent and reliable health marker in childhood and adolescence [6]

Age and sex differences in PF are well known. For example, Ortega et al. [7] studied adolescent boys and girls (12–17 years old) from 10 European countries and showed greater PF levels in boys than in girls (except for flexibility), as well as increased mean fitness with increasing age. Additionally, Bohr et al. [8] investigated the role of socioeconomic status (SES) on PF among US adolescents, and reported that girls in the low SES group were less fit than girls with higher SES; whereas for boys this relationship was not evident. Furthermore, in Swedish adolescents aged 14–15 years, Ekelund et al. [9] found positive associations between cardiorespiratory fitness and physical activity and biological maturation and a negative association with body fat. Despite the expectation of sociodemographic influences on PF expression, we only found one study [10] that investigated how children’s PF development across time was related to age and sex, as well as school grade, place of residence, paternal education and maternal education, i.e., sociodemographic factors. Data showed that most of the observed differences in PF were mainly accounted for by age and sex; additionally, children from the suburbs were more physically fit than those from urban areas.

The available research with twins and nuclear/extended families has indicated that genetic factors are responsible for a substantial portion of the total variation in PF at the population level. For example, Maes et al. [11] investigating 10-yr-old twin pairs and their parents from the Leuven Longitudinal Twin Study found that, in general, MZ twins were more similar, as described by intraclass correlations (ρ), in their motor performance than DZ twins (MZ, ρ = 0.46–0.83; DZ, ρ = 0.28–0.54). Recently, Schutte et al. [12] also reported higher correlations in MZ twins (ρ = 0.34–0.79) than DZ twins (ρ = 0.28–0.54) in vertical jump, handgrip strength, balance and flexibility. In addition, Pérusse et al. [13] examined familial aggregation in aerobic power, muscular endurance, and strength and showed that in all PF components, siblings’ correlations were higher than spousal and parent-child correlations. Furthermore, Lortie et al. [14] also investigated the familial similarity in maximal aerobic power and reported higher spousal and sibling resemblance (ρ = 0.34 and ρ = 0.33, respectively) than parent-child resemblance (ρ = 0.19).

Available data on PF among siblings is somewhat scarce, and we were able to find only eight papers on the topic, and the results were not consistent. For example, Malina & Mueller [15] used US data to show that brother-brother pairs (ρ = 0.46) were more similar than brother-sister (ρ = 0.24) and sister-sister pairs (ρ = 0.19) in their muscular strength. On the contrary, Pawlak [16] reported higher correlations in Polish adolescent sister-sister pairs (ρ = 0.44) than brother-brother (ρ = 0.25) or brother-sister pairs (ρ = 0.21) for grip strength.

Siblings share, on average, 50% of their genes identical-by-descent, and also have a common familial environment [17]. However, taking into account differences in their age and sex, as well as in their physical growth and motor development trajectories may provide more precise estimates of common and unique environmental variation in the expression of their health-related PF [18]. Investigating sibling resemblance in the expression of this complex construct, i.e., studying more than one child per family, may provide important clues regarding the separate and joint effects of sociodemographic, behavioural, or genetic factors on youth differences and/or similarities in their PF. Additionally, they may provide relevant indications when designing optimal physical activity intervention programs to combat the rise of obesity incidence [19] and decline in health-related PF levels [20].

Using the multilevel model as a statistical framework [21] as well as the Bouchard & Shephard PF template [1], the present study aims: to (1) investigate the separate and joint effects of biological, behavioural and sociodemographic characteristics in intra-pair sibling similarities in PF, (2) and estimate sibling resemblance in health-related PF components. The following hypotheses were posited: (1) biological, behavioural and sociodemographic characteristics are significantly associated with siblings’ health-‘related PF, and (2) sibling resemblance is higher in same-sex sibs than in opposite sex siblings for all PF components.

## Methods

### Study participants

Data are from the Portuguese Sibling Study on Growth, Fitness, Lifestyle and Health, which aims to investigate the physical growth, body composition, PF, physical activity, metabolic syndrome, and health behaviours of a large cohort of siblings. Children and adolescents, aged 9 to 20 years, were recruited in schools from the north and central regions of mainland Portugal, and were invited to freely participate in the project with their siblings and parents, since they were also previously called to be part of the Portuguese Healthy Family Study [22]. The project was approved by the Ethics Committee of the University of Porto, as well as by school authorities. Following their approval, all identified siblings from the ~8000 students enrolled in selected schools were invited to participate in the study. From 1376 identified/invited siblings, about 80% (response rate) agreed to take part in the study. Thus, a total of 1101 biological siblings (525 females and 576 males) from 540 nuclear families (519 pairs and 21 triplets) were sampled. Parents or legal guardians provided written informed consent.

### Anthropometry

Height and weight were measured using standardized protocols established by the International Society for the Advancement of Kinanthropometry [23]. Height was measured with a portable stadiometer (Holtain, UK) and weight with a portable bioelectrical impedance scale (TANITA BC- 418 MA Segmental Body Composition Analyzer Tanita Corporation, Japan). Body mass index (BMI) was computed using the standard formula (BMI = weight (kg)/(height (m))^2^).

### Maturity offset

Biological maturation was estimated with the maturity offset procedure, proposed by Mirwald et al. [24], which estimates the distance, in decimal years, each subject is from age at peak height velocity (PHV). A positive (+) maturity offset represents the number of years the participant is beyond PHV, whereas a negative (−) maturity offset represents the number of years the participant is before PHV.

### Health-related physical fitness

Using the Bouchard & Shephard health-related PF model comprising morphological (waist circumference and body fat percentage), muscular (strength and power), motor (speed and agility) and cardiorespiratory (aerobic capacity) components, all siblings underwent a systematic assessment comprised of the following tests:

Morphological component: Body fat percentage (%BF) was estimated using a portable bioelectrical impedance scale (Tanita BC-418 MA segmental body composition analyser, Tanita Corporation, Japan). Waist circumference (WC), anatomically identified as the smallest circumference between the lowest rib and the superior border of the iliac crest, was measured, in cm, with a non-elastic tape (Sanny, American Medical of Brazil, São Paulo, Brazil).

Muscular component: Muscular strength, namely static strength, was assessed with the grip strength (GS) test using a hand dynamometer (Takei Digital Grip Strength Dynamometer, Model T.K.K.5401, Tokyo, Japan), and the result was recorded in kgf. Muscular power was obtained with the standing long jump test (SLJ), and results were recorded in centimetres (cm).

Motor component: Speed was assessed with the 50 yard dash (50yd), and agility was marked with the shuttle-run (SHR); time was recorded in seconds (s).

Cardiorespiratory component: Aerobic capacity was estimated with the 1-mile run/walk test (1-MR), and the result was recorded in minutes (min).

### Physical activity

Total physical activity (TPA) was estimated with the Baecke questionnaire [25], a reliable and valid instrument [26] that describes three basic PA domains: school PA (questions related to sitting, standing, walking, lifting and sweating during school), leisure-time PA (questions associated with mode of transportation to school and time spent watching TV, walking and cycling) and sport participation (frequency of practice and sweating during sport practice). The Portuguese version of this questionnaire is widely used in children and youth [27] as well as in family studies [22]. The TPA score is obtained from the unweighted sum of the three domains. For each domain, each score ranges from 1 (minimal) to 5 (maximal), such that the TPA score varies between 3 and 15. All participants answered the questionnaires during their physical education classes, under the supervision of their physical education teacher as well as by a trained research team member.

### Screen time

Participants answered questions related to time spent watching television (TV), the response options for both questions, were: (1) <30 minutes; (2) 30 minutes-1 hour; (3) 1 hour-1:30 hours; (4) 1:30 hours-2 hours; (5) >2 hours). It is important to note that techniques to assess sedentary behaviours vary marked in the literature, and the use of screen time as a marker of sedentary behaviour in children is by far the most common. Self-reported information about sedentary behaviour has been commonly used in studies from different countries and/or cultures [28–31] as well as in studies with Portuguese samples [32, 33]

### Socioeconomic status

Socioeconomic status (SES) was assessed by asking participants about their parents’ occupations. The occupation was categorized into ten groups (from 0–9) according to the Portuguese National Classification of Occupations (2010), where group 0 is the highest SES and group 9 is the lowest. Categories are as follows: (0) armed forces (1) central administration/politicians and executive directors; (2) specialists of intellectual and scientifically activities; (3) technicians and intermediate level jobs; (4) back-office jobs; (5) security, seller and individual services; (6) farmer and qualified workers of farm, fish and forest; (7) industry and building qualified jobs: (8) machine and equipment operators; and (9) nonqualified jobs.

### Data quality control

To ensure data quality, the following procedures were used: (1) training of all team members by experienced researchers of the Kinanthropometry Laboratory of the Sports Faculty, University of Porto, Portugal; (2) conducting random retests on each assessment day. The technical error of measurement (TEM) for anthropometric measurements and body composition variables were 0.1 cm for height and WC, 0.1 Kg for weight, and 0.4% for %BF. Reliability calculations via ANOVA-based intraclass correlation coefficients (R) for all physical fitness tests were as follows: 1-MR, R = 0.97; SLJ, R = 0.95; SHR, R = 0.93; 50yd, R = 0.95; GS, R = 0.94.

### Statistical analysis

Basic exploratory (data check for outliers and normality) and descriptive statistics (mean±standard-deviations) were computed in IBM-SPSS 21. Given the skewed distribution in GS, a log transformation was used to normalize it. A multilevel model implemented in STATA 14 software was used given the data clustering–individuals nested within siblings pairs. In the analyses, models were estimated for each individual marker of PF using a set of covariates: age, age^2^, maturity offset, TPA, TV, SES, TPA-by-SS interaction, TPA-by-BS interaction, Age-by-SS interaction, and Age-by-BS interaction. Covariates were centered at their respective means as advocated [34]. Using a statistical approach developed by Hedeker et al., [35] that expands beyond the classical multilevel model, we estimated separate within and between siblings’ variances, and therefore separate intraclass correlations (ρ) with corresponding 95% confidence intervals (95%CI), for the three sibling types [brother-brother (BB), sister-sister (SS) and brother-sister (BS)]. Unadjusted, partially adjusted (age and age^2^), and fully adjusted (for all covariates mentioned above) intraclass correlations were computed. In all models, the BB pair served as the reference category. All parameters were simultaneously estimated using maximum likelihood [36].

## Results

Descriptive statistics for all PF components, demographic and behaviour indicators are provided in Table 1. On average, sibling pairs have similar chronological ages, but their mean age differences differ somewhat. Further, BB pairs are the tallest as well as the heaviest, have the highest WC on average, but lower %BF than BS and SS pairs. For the remaining PF components, BB pairs are stronger (GS and SLJ) and they performed better in the motor and cardiorespiratory components (50yd, SHR, and 1-MR) than BS and SS pairs. Mean SES and time spent watching TV is similar across sibling-types, but BB pairs are slightly more physically active.

**Table 1:** Descriptive statistics (means and standard deviations (SD)) for siblings pairs.

	Brother–Brother (n = 317)		Sister–Sister (n = 269)		Brother–Sister (n = 515)	
Variables	Mean	SD	Mean	SD	Mean	SD
Age (years)	13.5	2.0	13.3	2.1	13.3	2.0
Age difference (years)	2.0	1.6	2.4	1.8	2.5	1.6
Height (cm)	158.6	13.0	154.0	8.8	155.6	11.0
Weight (kg)	53.3	15.8	49.4	11.5	50.4	12.8
Body mass index (kg/m^2^)	20.8	4.0	20.7	3.8	20.5	3.6
**Morphological component**						
Waist circumference (cm)	70.4	9.9	66.0	8.0	67.7	8.1
%BF	19.9	6.5	26.5	5.7	23.3	6.9
**Muscular component**						
Hand grip strength (Kg^f^)	28.4	9.7	22.6	5.4	25.1	8.1
Standing long jump (m)	168.0	33.6	136.8	24.2	151.4	31.8
**Motor component**						
50 yard dash (s)	7.9	1.1	8.6	0.9	8.3	1.1
Shuttle-run (s)	11.1	1.8	12.1	1.7	11.4	1.8
**Cardiorespiratory component**						
1-mile run/walk (min)	8.6	2.1	10.4	2.2	9.3	2.2
**Demographic indicator**						
Socioeconomic status	5.7	2.2	5.5	2.5	5.9	2.2
**Behavioral indicators**						
Physical Activity	8.0	1.3	7.4	1.2	7.7	1.2
TV/day	2.8	1.4	2.7	1.2	2.7	1.3

: n = total number of subjects by sibling type.

Multilevel analysis results are presented in Table 2. For the morphological component, averages for BB pairs are 18.40±0.76% and 68.58±0.95 cm for %BF and WC (cm), respectively. For both %BF and WC, significantly lower values were observed with higher age (β = -2.33±0.23; β = -0.84±0.32), and with greater biological maturity (β = 1.52±0.20; β = 2.68±0.25). TPA, SES and TV did not significantly associate with these components. Sister-sister and BS pairs have significantly higher %BF (β = 6.49±0.68; β = 2.97±0.60) and lower WC (β = -4.48±0.92; β = -3.02±0.73) than the BB pairs. Total physical activity did not significantly interact with siblings’ in terms of %BF or WC, however age-by-siblings’ did for %BF; for SS and BS pairs higher age was associated with higher mean %BF levels as compared to BB pairs.

**Table 2:** Parameter estimates and variance components for each physical fitness test.

	Body fat (%)	Waist circumference (cm)	Hand grip (Log kg^f^)	Standing long jump (cm)	50 yard dash (s)	Shuttle-run (s)	1-mile run/walk (min)
Fixed Effects	Estimate±SE	Estimate±SE	Estimate±SE	Estimate±SE	Estimate±SE	Estimate±SE	Estimate±SE
Intercept (BB)	18.40±0.76^***^	68.58±0.95^***^	3.28±0.03^***^	166.74±2.78^***^	7.96±0.11^***^	10.84±0.20^***^	9.07±0.21^***^
SS	6.49±0.68^***^	-4.48±0.92^***^	-0.17±0.03^***^	-19.06±2.55^***^	0.30±0.10*	0.46±0.19*	0.65±0.22^**^
BS	2.97±0.60^***^	-3.02±0.73^***^	-0.09±0.02^***^	-8.19±2.08^***^	0.11±0.08^ns^	0.07±0.15^ns^	0.08±0.15^ns^
Age	-2.33±0.23^***^	-0.84±0.32^**^	0.08±0.01^***^	6.34±1.00^***^	-0.07±0.04^ns^	-0.01±0.07^ns^	-0.17±0.08*
Age^2^	0.07±0.04^ns^	-0.02±0.05^ns^	-0.008±0.001^***^	-0.54±0.15^**^	0.02±0.01^**^	0.03±0.01*	0.01±0.01^ns^
Maturity offset	1.52±0.20^***^	2.68±0.25^***^	0.06±0.01^***^	3.78±0.80^***^	-0.17±0.03^***^	-0.15±0.06^**^	-0.01±0.06^ns^
TPA	0.09±0.23^ns^	0.28±0.35^ns^	0.01±0.01^ns^	-0.12±1.04^ns^	0.06±0.04^ns^	0.16±0.07*	-0.13±0.08^ns^
TV	0.29±0.16^ns^	0.05±0.20^ns^	0.003±0.006^ns^	0.05±0.61^ns^	-0.01±0.02^ns^	0.02±0.04^ns^	0.07±0.05^ns^
SES	0.08±0.10^ns^	0.19±0.12^ns^	-0.000±0.003^ns^	-0.78±0.36*	0.02±0.01^ns^	0.09±0.03^**^	0.00±0.028^ns^
TPA*SS	-0.38±0.40^ns^	-0.77±0.56^ns^	-0.001±0.015^ns^	2.34±1.61^ns^	-0.17±0.07^**^	-0.36±0.12^**^	-0.17±0.14^ns^
TPA*BS	-0.55±0.36^ns^	-0.34±0.45^ns^	-0.01±0.01^ns^	2.50±1.39^ns^	-0.13±0.05*	-0.30±0.98^**^	-0.19±0.11^ns^
Age*SS	1.85±0.25^***^	0.21±0.34^ns^	-0.05±0.01^***^	-5.44±1.02^***^	0.01±0.04^ns^	0.02±0.08^ns^	0.10±0.09^ns^
Age*BS	0.99±0.25^***^	0.29±0.31^ns^	-0.02±0.01*	-1.93±0.97*	0.01±0.04^ns^	-0.01±0.07^ns^	-0.05±0.07^ns^
%BF	——	——	-0.001±0.001^ns^	-2.08±0.12^***^	0.07±0.01^***^	0.08±0.01^***^	0.16±0.01^***^
Variance components (σ^2^)							
Between siblings’	Estimate±SE	Estimate±SE	Estimate±SE	Estimate±SE	Estimate±SE	Estimate±SE	Estimate±SE
BB	21.700±3.736	27.345±6.389	0.003±0.001	127.527±45.925	0.176±0.065	0.823±0.237	0.306±0.254
SS	14.437±3.319	32.760±6.657	0.005±0.001	208.041±48.458	0.262±0.078	1.097±0.283	1.981±0.433
BS	0	7.038±3.551	0	46.365±38.218	0.091±0.053	0.310±0.204	0.421±0.208
Within siblings’							
BB	14.619±1.812	38.337±4.840	0.006±0.001	387.042±47.825	0.542±0.067	1.749±0.218	2.570±0.319
SS	16.446±2.268	27.266±3.816	0.004±0.001	236.777±33.273	0.491±0.068	1.529±0.213	2.005±0.280
BS	44.557±3.160	41.324±4.216	0.008±0.001	479.695±49.310	0.647±0.066	2.405±0.249	2.472±0.250

*p<0.05

**p<0.01

***p<0.001

ns = non-significant.

For the muscular component, the mean performance of BB pairs is 3.28±0.03 for GS (log-units) and 166.74±2.78 for SLJ (cm). Older subjects are stronger (β = 0.08±0.01; β = 6.34±1.00) in both tests, as are more mature children (β = 0.06±0.01; β = 3.78±0.80). Higher %BF is linked with poorer performance (β = -2.08±0.12) in SLJ, but not in GS; TPA and TV do not significantly associate with muscular performance. SS (β = -0.17±0.03; β = -19.06±2.55) and BS (β = -0.09±0.02; β = -8.19±2.08) have lower values than BB pairs on both tests. SES was significantly related with lower values in SLJ (β = -0.78±0.36), but not in GS. Total physical activity did not significantly interact with siblings’ for both strength tests, but age-by-siblings did, such that in SS and BS pairs, higher age showed lower mean GS and SLJ than in BB pairs.

The motor component averages for BB pairs are β = 7.96±0.11 (50yd dash in s) and β = 10.84±0.20 (SHR in s). More mature subjects performed better in both tests (β = -0.17±0.03 and β = -0.15±0.06 for 50yd and SHR respectively). Those with higher %BF performed worse (β = 0.07±0.01 and β = 0.08±0.01 for 50yd and SHR, respectively). Age and TV did not significantly associate with the motor components.

TPA and SES showed no association with 50yd, but are significantly linked with lower SHR performance (β = 0.16±0.07; β = 0.09±0.03). Age-by-siblings’ did not interact with the motor components. Yet, TPA-by-siblings’ did, where in both SS and BS higher TPA was associated with higher means in both tests than BB pairs. SS pairs show lower performance levels than BB pairs in both tests (β = 0.23±0.10 and β = 0.46±0.19 for 50yd and SHR, respectively). BS pairs does not differ from BB pairs (p>0.05).

Finally, in the cardiorespiratory component, BB pairs´ 1-Mile run/walk average is 9.07±0.21 (min): SS pairs need more time (β = 0.65±0.22) to cover the distance than BB pairs, but not BS pairs (p>0.05); also, older subjects are better performers (β = -0.17±0.08). Of all covariates, only %BF is associated with the 1-MR–those with higher %BF require more time to cover the distance (β = 0.16±0.01).

Unadjusted, partially adjusted and fully adjusted intraclass correlations are provided in Table 3. In most cases, the inclusion of covariates had only a minimal influence on the magnitude of the intrapair correlations. However, in some cases the correlations were affected by the inclusion of covariates. For example, the BB unadjusted correlation for 1MR was 0.35 which dropped to 0.11 after the inclusion of all covariates. In general, the correlations for BS pairs were much lower than for the same-sex pairs across the board, irrespective of the inclusion of additional covariates.

**Table 3:** Intraclass correlation coefficients (ρ) and (95% CI) for each physical fitness test.

	Body fat (%)	Waist circumference (cm)	Hand grip (Log kg^f^)	Standing long jump (cm)	50 yard dash (s)	Shuttle-run (s)	1-mile run/walk (min)
BB							
Unadjusted	0.54 (0.43–0.64)	0.41 (0.29–0.54)	0.21 (0.10–0.39)	0.29 (0.14–0.50)	0.43 (0.32–0.56)	0.44 (0.32–0.56)	0.35 (0.23–0.50)
Adjusted*	0.54 (0.43–0.65)	0.43 (0.32–0.59)	0.27 (0.15–0.44)	0.33 (0.20–0.48)	0.39 (0.27–0.53)	0.42 (0.30–0.55)	0.37 (0.25–0.51)
Fully adjusted**	0.60 (0.48–0.70)	0.42 (0.28–0.56)	0.30 (0.16–0.48)	0.25 (0.12–0.44)	0.25 (0.12–0.44)	0.32 (0.19–0.43)	0.11 (0.02–0.42)
SS							
Unadjusted	0.42 (0.29–0.56)	0.40 (0.27–0.55)	0.30 (0.17–0.47)	0.28 (0.15–0.46)	0.43 (030–0.57)	0.43 (0.30–0.57)	0.46 (0.33–0.59)
Adjusted*	0.42 (0.29–0.56)	0.46 (0.34–0.60)	0.46 (0.33–0.59)	0.33 (0.20–0.50)	0.32 (0.19–0.49)	0.48 (0.35–0.61)	0.46 (0.33–0.59)
Fully adjusted**	0.47 (0.33–0.61)	0.55 (0.41–0.67)	0.54 (0.40–0.69)	0.47 (0.32–0.62)	0.35 (0.20–0.53)	0.41 (0.27–0.58)	0.50 (0.36–0.64)
BS							
Unadjusted	0	0.18 (0.09–0.32)	0	0	0	0	0
Adjusted*	0	0.28 (0.18–0.41)	0.08 (0.02–0.29)	0	0.06 (0.01–0.35)	0.03 (0.00–0.66)	0
Fully adjusted**	0	0.15 (0.05–0.34)	0	0.09 (0.02–0.36)	0.12 (0.04–0.34)	0.11 (0.03–0.35)	0.14 (0.05–0.34)

*adjusted for age and age^2^

** adjusted for all covariates.

## Discussion

Biological, behavioural and sociodemographic characteristics are expected to be associated with PF components in different ways. For example, greater chronological age has consistently been associated with better PF during childhood and adolescence, although with varying degrees of magnitude [18]. In the present study, the maturity offset was found to be positively associated with siblings´ muscular and motor components, negatively with morphological fitness, and had no significant effect on the cardiorespiratory component. Previous relationships between chronological and biological ages and PF have been reported, but it is not always easy to clearly separate their unique contributions as well as their links to changes in body size [18]. For example, Welk et al. [37] using chronological age and Jones et al. [38] using biological age both showed positive associations with PF, while other studies based on chronological age reported the contrary [39]. Further research is required to better delineate the roles of chronological and biological age on PF levels in childhood.

As expected, %BF was negatively associated with PF in the present study, except for GS (not statistically significant). These results support previous reports using non-sibling data [18]. For example, Moliner-Urdiales et al. [40] showed that Spanish adolescents with lower %BF were more physically fit. In a different vein, behavioural characteristics (TV and TPA) were not strongly associated PF components in the present study. A previous study among 11 to 18 year old children showed that, among boys, high TV viewing time (≥2 h/day) was positively related with their WC, and that high total screen time (>3 h/day) was positively associated with their WC and BMI; no such associations were found in girls [41]. Moreover, Dencker et al. [42] reported that cardiorespiratory fitness was positively correlated with vigorous physical activity but not with moderate physical activity in Swedish youth, and two studies in youth from the USA and Spain, Bai et al. [43], Ara et al. [44] showed positive associations between physical activity and PF. Overall, there is some evidence that physical activity is related to different PF components in children; however more research is required to better determine the magnitude and direction of the associations.

In the present study, SES was negatively associated with SLJ and SR. Previous research on siblings´ PF did not consider the putative effect of SES. However, Mutunga et al. [45] examined the relationship between SES and VO_2max_ in a large sample of adolescents from Northern Ireland and reported that those with higher SES had higher cardiorespiratory fitness. Similarly, Jimenez Pavón et al. [46] using data from different European countries showed that boys and girls with higher SES were also more physically fit in muscular, motor and cardiorespiratory components. In sum, it is important to consider SES as a covariate when interpreting familial aggregation of PF components. The interpretation of similarities among twins, siblings or nuclear family members in health-related PF is always conditioned by several issues, namely: (1) differences in sample size across studies, (2) population of origin/ethnicity, (3) differences in measurement protocols for PF assessments, (4) data reliability, (5) differences in statistical approaches, and (6) differences in covariates included in the models.

In general, same sex siblings´ morphological fitness (%BF and WC) were systematically more alike, whereas opposite sex siblings showed no resemblance in %BF, and only moderate similarity in in WC. It is also possible that unique developmental histories of each member of the opposite sex in opposite-sex sibling pairs during his/her physical growth, biological maturation, exercise habits and food consumption may explain the absence of similarity in %BF, and low similarity in WC. This sexual dimorphism is pervasive in many biological traits [47–49]. Contrary to our findings, previous studies showed lower sibling similarity in morphological traits than we have shown. For example, Katzmarzyk et al. [50] reported a intra-pair sibling correlation of 0.25 for WC and Rice et al. [51] presented an intrapair sibling correlation of 0.32 for %BF in the HERITAGE Family Study. However, in these studies, siblings were not separated by type (BB, SS and BS pairs) and their analysis only adjusted for age and sex. Taken together, these positive correlations suggest that shared genetic and sociodemographic factors play important roles in sibling similarity. To date, there are some specific genes that have been linked to morphological fitness traits. Rankinen et al. [52] reviewed the available literature on the human obesity gene map and reported that WC was associated with biological markers in 10 genes. For example, Robitaille et al. [53], Kim et al., [54] and Fornage et al., [55] found associations with PPAR- ρ and WC; PPAR- ρ is a nuclear receptor that regulates adipocyte differentiation and possibly lipid metabolism and can therefore be a key regulator of fat storage. Recently Lu et al., [56] conducted a genome-wide association study (GWAS) for %BF in 100,716 individuals, and identified 12 loci, eight of which were previously associated with overall adiposity (BMI, %BF) and four were novel in their associations with %BF.

For muscular fitness (GS and SLJ), with increasing covariate adjustments, SS pairs were more alike in both tests than BB pairs, while BS similarity is very low or absent. These differences could partially be explained by sex-differences in physiological, biochemical and hormonal mechanisms associated with muscular strength [18, 57], as well as by differences in body size since we did not scale their performance (see Asmussen [58]; Nevil et al., [59]). In any case, the higher resemblance in same-sex siblings may indicate biological, behaviour and sociodemographic characteristics, as well as genetic influences on these phenotypes [11, 60]. Mozambican [61] and Polish [16] data also showed that SS pairs (ρ = 0.19; ρ = 0.44) were more similar than BB (ρ = 0.09; ρ = 0.25) and BS (ρ = 0.02; ρ = 0.21) in GS. For SLJ, correlations were different across studies: Saranga et al., [61] reported the highest resemblance in SS (ρ = 0.44) followed by BS (ρ = 0.39) and BB (ρ = 0.23), while Pawlak [16] showed the highest resemblance in BB pairs (ρ = 0.29) but similar results in SS pairs (ρ = 0.16) and BS pairs (ρ = 0.16), although in these studies the analysis only adjusted for age and sex. The precise location of responsible genes for muscular fitness is still under scrutiny. GWAS data as well as association studies with candidate genes are scarce and inconsistent, respectively. Two recent reviews examined candidate genes for muscular strength and categorized them according to either their involvement in the structural muscle function or their influencing role in muscle physiology [62, 63]. For example, the MSTN K153R polymorphism is associated with the ability to produce peak power during muscle contractions, as assessed with jump tests, in young non-athletic men [64]. We only found one study that tried to identify single nucleotide polymorphisms (SNPs) associated with GS in middle-aged to older adults using GWAS [65]. However, no genome-wide significant results were observed.

For the motor fitness component, BB and SS pairs demonstrated greater similarities than BS pairs. The unique environment, i.e., life history idiosyncrasies of each member of the sibling pair may help in the interpretation of dissimilarities between them. On the other hand, Saranga et al., [61] reported greater familiality in Mozambican BB pairs (ρ = 0.13) than SS (ρ = 0.04) and BS (ρ = -0.02) in the SR test. Differences between these two studies may be due to ethnicity as well as diverse sociodemographic conditions. Nonetheless, our results indicate that the motor performance component is likely influenced by genetic and sociodemographic characteristics, although we were not able to find a study that explicitly examined the role of any putative gene, or genes, in the phenotypic expression of this PF component. The ACTN3 gene has been associated with performance in sprint athletes. For example, Yang et al., [66] studied athletes and non-athletes and reported that male and female elite athletes had significantly higher frequencies of the 577R allele than the non-athletes. Further, Kim et al., [67] examined the association between the distribution of ACTN3 genotypes and alleles in muscle power, speed, and strength-oriented athletics and showed that only the speed-oriented athletes had significant differences in the frequency distributions of the ACTN3 XX genotype from that of the controls.

For cardiorespiratory fitness, the unadjusted and adjusted ρ values showed that SS pairs display greater resemblance than BB and BS. These results may indicate that females are more prone to display genetic and/or shared environmental putative effects than males. Previous reports with Mozambican [61] and Portuguese [68] siblings also showed greater familiality in SS pairs than in BB or BS pairs. However the cardiorespiratory component is a highly complex phenotype in terms of its physiologic and biochemical mechanisms. It is thus rather difficult to signal the presence of relevant genes in the expression of this component, since there is no single gene to determine cardiorespiratory fitness [69]. Although we did not find any association studies with candidate genes in the phenotypic expression of the 1-mile run, or any other marker of cardiorespiratory endurance with children and adolescents, yet several studies based on other phenotypes were previously reviewed using adult samples [70], and more than 30 genes have been recently found, from more than five physiological areas (regulating hormones, muscle metabolism, lipid metabolism, growth factors, cellular mediators and others) [69]. Although highly debatable [71], a polymorphism of the angiotensin I-converting enzyme (ACE) gene has been associated with metabolic efficiency, specifically the D-allele with an exaggerated response to training, and the I-allele with the lowest cardiac growth response. In light of the I-allele association with endurance performance, it seems likely that other regulatory mechanisms exist [72].

Given the use of a large sample of siblings, a large age range covering childhood and adolescence, and extended PF assessment, this allows us to extend our conclusions to single children and adolescents enrolled in the same schools and living in similar environments. Since PF is an important marker of health in children and adolescents [6] physical activity intervention program designers should consider targeting families given the importance of family members as role models which may have an influence on developing PF levels in children. Additionally, since different sex siblings are more dissimilar, programs to enhance PF levels in childhood should also consider sex-specific strategies. Although it is well acknowledged that individual responses to a physical activity intervention program is highly variable and may have a genetic basis [73] we anticipate that research involving siblings will enhance our understanding of the interplay of nurture and nature in their PF similarity/dissimilarity during childhood and adolescence. This information is currently lacking.

Notwithstanding the relevance of these results, our study has limitations: (1) the sample does not cover all Portuguese regions, and this limits the generalizability of the results to all Portuguese children and adolescents; (2) the use of a questionnaire to obtain information about PA and TV viewing is prone to errors, even when it is collected under controlled conditions. However, the use of self-report instruments to obtain this information is common [32, 74, 75]. Additionally, the Baecke questionnaire is regularly used in Portuguese studies with highly reliable results [22, 76–78]. This study also has several strengths, including: (1) the use of a broad approach of health-related physical fitness with siblings, (2) the large sample size of siblings (3) the examination of important time windows in children’s growth and development, (4) the use of standard measurement protocols and highly reliable data, and (5) the use of a novel, multilevel statistical methodology.

## Conclusions

In conclusion, our results revealed that siblings’ PF similarity varies according to its components. Same-sex siblings demonstrate greater resemblance in all PF components. Additionally, older and more mature subjects are better performers. More physically active subjects do not perform better than those with lower physical activity levels, except in the shuttle run where the most active youth tend to have worse performance; time spent watching TV did not show any association with any physical fitness component, but higher SES was negatively associated with standing long jump and shuttle run performance. Taken together these results reinforce the idea that PF it is not only the end result of genetic endowments, but also of shared and unique environmental factors which can act alone or in association with genes, modulating their expression.
